# Patient education materials to implement choosing wisely recommendations for internal medicine at the emergency department

**DOI:** 10.1136/bmjoq-2020-000971

**Published:** 2021-02-04

**Authors:** Bart J Laan, Willemijn B Huiszoon, Frits Holleman, Marja A Boermeester, Karin A H Kaasjager, Suzanne E Geerlings

**Affiliations:** 1 Department of Internal Medicine, Division of Infectious Diseases, Amsterdam University Medical Center, University of Amsterdam, Amsterdam, The Netherlands; 2 Department of Internal Medicine, University Medical Center Utrecht, Utrecht, The Netherlands; 3 Department of Internal Medicine, Amsterdam University Medical Center, University of Amsterdam, Amsterdam, The Netherlands; 4 Department of Surgery, Division of Infectious Diseases, Amsterdam University Medical Center, University of Amsterdam, Amsterdam, The Netherlands

**Keywords:** patient-centred care, patient education, quality improvement, shared decision making

## Abstract

**Background:**

Choosing Wisely aims to reduce low-value care to improve quality and lower costs. In the Netherlands, this campaign offers three recommendations for internal medicine applicable in emergency departments (EDs): (1) do not place an indwelling urinary catheter in non-critically ill patients who can void; (2) do not order plain abdominal radiographs in patients with acute abdominal pain; and (3) discuss whether treatment limitations are needed. This quality improvement project aims to increase the implementation of the recommendations by patient information leaflets.

**Methods:**

In a prospective before–after study, we collected data every other week during baseline and intervention periods (both 7 months) in two university medical centres. The primary outcomes were the adherence rates to the recommendations.

**Results:**

805 patients visited the EDs for internal medicine, of whom 391 (48.6%) were hospitalised. Only 153 (19%) patients received the information leaflet. We found no change in implementation rates of the recommendations after the introduction of the patient information leaflet. In the baseline period, 28 patients received a urinary catheter, of whom 5 (17.9%) had no appropriate indication, compared with 4 (25.0%) of 16 patients in the intervention period (p=0.572). Unnecessary abdominal X-ray occurred once in the baseline period and not in the intervention period. Treatment limitations were not reported in 13 (6.5%) of 200 hospitalised patients in the baseline period, and in 17 (8.9%) of 191 patients in the intervention period (p=0.373).

**Conclusions:**

Patient information leaflets did not increase the implementation of Choosing Wisely recommendations, which can be due to a high baseline rate and a poor dissemination of leaflets. Our ED seems not to be a practicable setting for dissemination of leaflets, since staff engagement was not possible due to high workload and shortage of qualified nursing staff in the Netherlands.

## Introduction

Low-value care, such as overuse and waste, is unlikely to benefit patients and could even harm them. To address low-value care, in 2012 the American Board of Internal Medicine Foundation developed Choosing Wisely, a physician-driven campaign to create conversations between physicians and patients about unnecessary tests, treatments and procedures.^1^ Choosing Wisely has spread worldwide to more than 20 countries, and since 2014 the Netherlands is participating.^2^ A main part of the Choosing Wisely Netherlands Campaign is the development of lists of evidence-based recommendations to improve healthcare and reduce costs. The Netherlands Association of Internal Medicine (NIV) also created a list of 10 Choosing Wisely recommendations with the support of scientific communities. The main goal was shared decision-making to provide each patient with the best treatment at the right moment. The list was developed by a bottom-up approach, through a survey via email to all members of the NIV asking them for any item to be proposed.

Early trends of seven Choosing Wisely recommendations in the USA showed only minimal benefits.^3^ Further, many clinical nuances for a lot of Choosing Wisely recommendations exist, and accurate measurement of these low-value care practices is challenging.^4^ The next step of Choosing Wisely was a shift from recommendation development towards implementation.^5^ Effective strategies to reduce low-value care are through interventions that engage patients in the physicians–patient interaction.^6^ Moreover, a systematic review found that multicomponent interventions for both patients and healthcare workers have the highest potential to reduce low-value care, but patient education alone could also be a low-cost intervention to change patients’ behaviour and reduce overuse.^7^

In the Netherlands, the 10 Choosing Wisely recommendations for internal medicine were evidence based, but published and communicated explicitly by the NIV. Some recommendations are implemented in clinical wards by quality improvement projects, such as the RICAT project to reduce inappropriate use of catheters and the implementation of the antibiotic checklist to optimise treatment of antibiotics, including prompt intravenous–oral switch.^8 9^ However, there was no further implementation of all recommendations with decision-supporting materials.

The recommendations are applicable not only in certain diseases, tests, treatments or procedures, but also in different hospital services (outpatient clinic, emergency department (ED), clinical wards and so on). A conversation between physicians and patients could be more difficult in the ED compared with other hospital services, since physicians and nurses have multiple simultaneous tasks with many interruptions in the ED.^10^ Furthermore, physicians and patients are generally meeting each other for the first time in the ED, and medical care is mostly acute and therefore stressful. Most patients will have to wait at least 1 hour for all tests results, and therefore could have time to read patient information about the recommendations, and afterwards start a conversation about unnecessary tests, treatments and procedures.

Three recommendations are applicable in the ED: (1) do not place an indwelling urinary catheter in non-critically ill patients who can void; (2) do not order plain abdominal or thoracic radiographs in patients with acute abdominal pain; and (3) discuss whether treatment limitations are needed when talking to patients about treatment options. Talking about treatment preferences and limitations is part of every treatment plan in the Netherlands, and a new discussion about treatment limitations should be held before each invasive procedure and admission. In the present project, we provided simple, low-cost educational materials for patients as part of regular care to implement the three Choosing Wisely recommendations for internal medicine in two EDs. We aimed to reduce the prevalence of the low-value care practices from the recommendations to less than 15%.

## Methods

**Figure 1:**
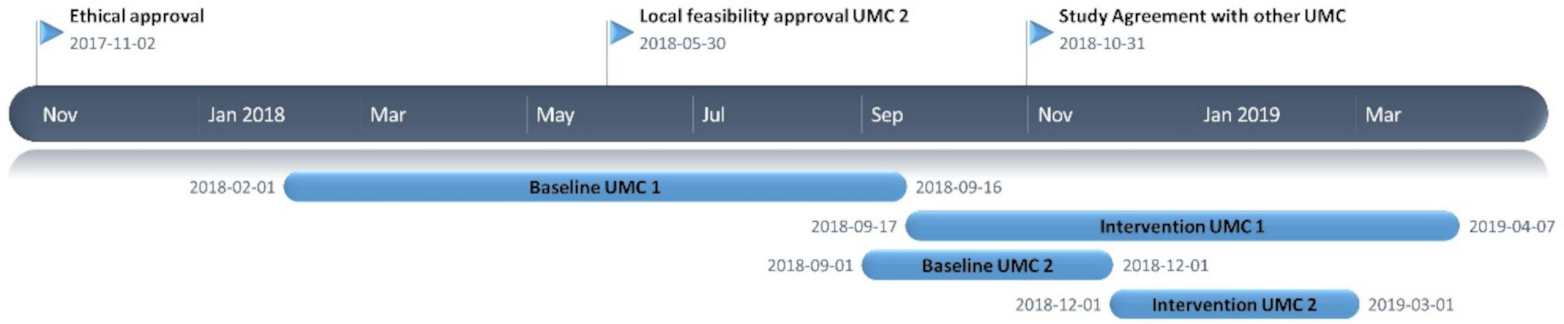
Timeline diagram.

We performed a prospective before–after quality improvement project in the EDs of two university medical centres in the Netherlands from 1 February 2018 to 3 April 2019. The first university medical centre is a 31-bed level 1 trauma centre with approximately 29 000 visits annually, and the second university medical centre is a 19-bed level 1 trauma centre with approximately 20 000 visits annually. Data were collected 1 day per week during a baseline period and an intervention period of each 3 months from all patients visited the ED for the department of internal medicine. The intervention period started after we introduced the intervention. Detailed information about the time periods of inclusion is provided in figure 1.

The Institutional Review Board of the Academic Medical Centre evaluated our project on 2 November 2017, and full review and individual informed consent were waived. Local feasibility was approved by the local ethics committees and board of directors of the participated hospitals. The results are reported in accordance with the Standards for Quality Improvement Reporting Excellence V.2.0 (SQUIRE 2.0) guidelines for reporting improvements in healthcare.^11^

### Recruitment of participants

We included patients that visited the ED for the department of internal medicine during an inclusion day, which was ones per week. Patients younger than 18 years of age were excluded. We collected patient characteristics and outcome data of the Choosing Wisely recommendations from electronic patient records of all (admitted and non-admitted) patients the day after the presentation at the ED. In addition, we planned to visit the admitted patients in the internal medicine wards to ask, if they had given verbal informed consent, whether they received, read and discussed the summary leaflet.

### Patient and public involvement

Patients or the public were not involved in the design, or conduct, or reporting or dissemination plans of our research.

### Intervention

A one side A4 summary leaflet of the Choosing Wisely recommendation relevant to the setting of the ED was distributed among patients visiting the ED for internal medicine. The summary leaflet highlighted the three recommendations, and we included a short description with background information in plain language. This summary leaflet was electronically accessible, and paper forms were available in the EDs. In both hospitals, the quality improvement teams consisted of a study coordinator, a resident internal medicine and the residency programme director. In one hospital, also a coordinating research nurse of the ED was added to the team. We planned to disseminate the leaflets by the nurse in the triage room as part of regular care. However, due to practical reasons, residents of internal medicine disseminated the leaflets. Physicians were instructed to discuss decision-making about the recommendations, if applicable, with their patients as in regular care. For recommendations 1, *Do not place an indwelling urinary catheter in non-critically ill patients who can void*, and 3, *Discuss whether treatment limitations are needed when talking to patients about treatment options*, the summary leaflet also referred to other patient information leaflets for further information. The summary and additional patient information leaflets were available in Dutch (online supplemental figures 1, 2 and 3). The readability of the leaflet was assessed by the Department of Patient Education and Counselling. For the already existing patient information leaflets, we checked that the information was completely up-to-date and no corrections were necessary.

10.1136/bmjoq-2020-000971.supp1 Supplementary data

10.1136/bmjoq-2020-000971.supp2 Supplementary data

10.1136/bmjoq-2020-000971.supp3 Supplementary data

### Outcomes

Outcomes measurements were trends in implementation of the three Choosing Wisely recommendations, namely, (1) percentage of inappropriate indications for insertion of a urinary catheter, (2) number of abdominal X-rays performed for acute abdominal pain and (3) percentage of hospitalised patients where treatment limitations were discussed and reported before 13:00 on the day after presentation at the ED. Appropriate indications for urinary catheter use were based on evidence-based recommendations.^12^ The result of the discussion about treatment limitation (yes/no limitations) was not important for the implementation of the third recommendation. Process measurements were the percentage of screened patients that received, read and discussed the patient summary leaflet.

### Data analysis

We calculated the sample size for recommendation 3, *Discuss whether treatment limitations are needed when talking to patients about treatment options*. A random sample of 14 patients in another hospital in the Netherlands showed that 30% of admitted patients had no reported treatment limitations. Based on the assumption of 15% absolute reduction, a sample size of 134 hospitalised patients per period (before and after the intervention) was necessary to achieve 80% power to detect a difference with a 0.05 two-sided significance level. Due to the study setting, we anticipated no dropouts and no missing data.

Categorical data were calculated as frequency and percentage, and continuous data as mean (SD) or median (range). We used unpaired t-tests or Mann-Whitney U tests for continuous variables and χ^2^ tests for categorical variables for comparisons of raw data. To adjust for confounders, we used bivariate logistic regression analyses for the possible confounders. Variables showing a difference of more than 10% in beta for baseline and intervention period were included in the multivariate logistic models to adjust for confounding. In addition, we evaluated the reported treatment limitations explorative. A two-sided p value <0.05 was considered significant without adjustment for multiple hypothesis testing. Descriptive analyses were performed using IBM SPSS Statistics, V.25.0.

## Results

**Figure 2:**
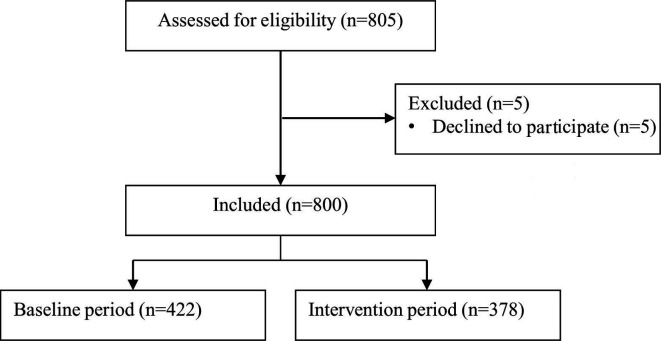
Trial profile.

Between 1 February 2018 and 7 April 2019, 805 patients visited the ED for the internal medicine in the two university medical centres. A total of 5 patients were excluded because permission to access their medical records was not obtained, 1 in the baseline period and 4 in the intervention period, resulting in 800 included patients (figure 2). In May 2018, we extended the baseline period of the first university hospital to reach the sample size, because at that moment we had not received approval of local feasibility for the second university hospital yet (figure 1). Primary endpoint data were available for all included patients. Baseline characteristics were similar between the periods, although patients in the intervention period had a lower Modified Early Warning System (MEWS) score (table 1). Further, 391 (48.9%) patients were hospitalised after visiting the ED.

**Table 1:** Baseline characteristics

	Baseline period (n=422)	Intervention period (n=378)	All (n=800)
Sex			
Male	216 (51.2%)	211 (55.8%)	427 (53.4%)
Female	206 (48.8%)	167 (44.2%)	373 (46.6%)
Age (years), mean (SD)	55.2 (18.8)	56.7 (19.2)	55.9 (19.0)
Hospital			
University hospital 1	268 (63.5%)	241 (63.8%)	509 (63.6%)
University hospital 2	154 (36.5%)	137 (36.2%)	291 (36.4%)
Specialism			
Gastroenterology	26 (6.2%)	25 (6.6%)	51 (6.4%)
Geriatrics	8 (1.9%)	17 (4.5%)	25 (3.1%)
Internal medicine	246 (58.3%)	220 (58.2%)	466 (58.2%)
Oncology	119 (28.2%)	97 (25.7%)	216 (27.0%)
Other	23 (5.5%)	19 (5.0%)	42 (5.3%)
Hospitalised 3 months before ED visit	112 (26.5%)	122 (32.3%)	234 (29.3%)
Outpatient care for internal medicine 1 year before presentation at the ED	300 (71.1%)	279 (73.8%)	579 (72.4%)
Nursing home resident	12 (2.8%)	10 (2.6%)	22 (2.8%)
Charlson Comorbidity Index^24^			
0	141 (33.4%)	125 (33.1%)	266 (33.3%)
1	58 (13.7%)	54 (14.3%)	112 (14.0%)
2	82 (19.4%)	92 (24.3%)	174 (21.8%)
≥3	141 (33.4%)	107 (28.3%)	248 (31.0%)
High MEWS score (total score ≥5 or any single physiological parameter scored +3)*	38 (9.0%)	17 (4.5%)	55 (6.9%)
Reason for emergency department visit			
Cardiovascular	30 (7.1%)	25 (6.6%)	55 (6.9%)
Endocrine	37 (8.8%)	22 (5.8%)	59 (7.4%)
Gastrointestinal	66 (15.6%)	72 (19.0%)	138 (17.3%)
Infectious	112 (26.5%)	104 (27.5%)	216 (27.0%)
Respiratory	37 (7.8%)	24 (6.3%)	61 (7.6%)
Nephrology	33 (7.8%)	21 (5.6%)	54 (6.8%)
Oncology or haematology	56 (13.3%)	64 (16.9%)	120 (15.0%)
Other	51 (12.1%)	46 (12.2%)	97 (12.1%)
Hospitalisation	200 (47.4%)	191 (50.5%)	391 (48.9%)

Data are n (%).

*P value<0.05.

ED, emergency department; MEWS, Modified Early Warning System.

After the introduction of our patient summary leaflet, no changes in the implementation of the three Choosing Wisely recommendations for internal medicine in the ED were found over the 14-month period (Table 2 and online supplemental figures 4 and 5). For recommendation 1, 5 (17.9%) of 28 patients who received a urinary catheter had no appropriate indication in the baseline period, and 4 (25.0%) of 16 patients in the intervention period (p=0.572). For recommendation 2, only one abdominal X-ray was performed for acute abdominal pain in the baseline period and none in the intervention period. For recommendation 3, treatment limitations were not reported in 13 (6.5%) of 200 hospitalised patients in the baseline period, and in 17 (8.9%) of 191 patients in the intervention period (p=0.373). The discussion for a possible treatment limitation was not reported in 41 (20.5%) of 200 hospitalised patients in the baseline period, and in 42 (22.0%) of 191 patients in the intervention period (p=0.719). Adjusted analyses for confounders showed similar results (online supplemental tables 1 and 2).

10.1136/bmjoq-2020-000971.supp4 Supplementary data

10.1136/bmjoq-2020-000971.supp5 Supplementary data

10.1136/bmjoq-2020-000971.supp6 Supplementary data

10.1136/bmjoq-2020-000971.supp7 Supplementary data

**Table 2:** Outcomes of implementation of the Choosing Wisely recommendations of internal medicine in the emergency departments

	Control period (n=422)	Intervention period (n=378)	P value
*1. Do not place an indwelling urinary catheter in non-critically ill patients who can void*			
Inappropriate indication for insertion of urinary catheters	5/28 (17.9%)	4/16 (25.0%)	0.572
*2. Don’t order plain abdominal X-rays in patients with acute abdominal pain*			
Abdominal X-rays performed for acute abdominal pain	1/26 (3.8%)	0/25 (0.0%)	>0.999
*3. Discuss treatment limitations when talking to patients about treatment options*			
No reported treatment limitations in hospitalised patients	13/200 (6.5%)	17/191 (8.9%)	0.373
No reported discussion of treatment limitations in hospitalised patients	41/200 (20.5%)	42/191 (22.0%)	0.719

Of the random subset of 113 patients from both hospitals, 68 were asked about the summary leaflet. The other 46 patients were not asked due to practical reasons, such as patient absent during evaluation, language barrier or patient admitted to a different ward or intensive care unit (ICU). Results showed that 13 (19.1%) of the 68 patients or their family received the summary leaflet, of which 9 (69.2%) read the summary leaflet. No patient read the additional patient information about urinary catheters, and seven patients read the additional information about treatment limitations. Of these, three patients stated that the information helped them in the discussion with the physician about possible treatment limitations.

There were 581 (72.6%) reported treatment limitations (including no limitations) in 800 patients, with 477 (59.6%) already reported before the presentation at the ED. We found that 323 (40.4%) had a reported (re-)evaluation of the treatment limitations. The specific limitations are presented in table 3. Most patients had a documentation of no limitations. If there was a documented limitation, most were do not resuscitate, do not start mechanical ventilation and do not admit to the ICU.

**Table 3:** Treatment limitations

Documentation of treatment limitations	Total (n=581)	
No treatment limitations	475 (81.8%)	
Treatment limitations		Not specifically reported
DNR	106 (18.2%)	0
No mechanical ventilation	66 (11.4%)	3 (0.5%)
No non-mechanical ventilation	62 (10.7%)	4 (0.7%)
No ICU (or CCU*)	54 (9.3%)	4 (0.7%)
No dialysis	2 (0.3%)	104 (17.9%)
No surgery	3 (0.5%)	103 (17.7%)
No blood products	5 (0.9%)	51 (8.8%)
No antibiotics	1 (0.2%)	104 (17.9%)
No invasive diagnostics	2 (0.3%)	104 (17.9%)

Data are n (%).

The specific limitations are presented only if they were reported explicitly.

*Specific reported no CCU in three patients.

CCU, cardiac care unit; DNR, do not resuscitate; ICU, intensive care unit.

## Discussion

Our educational materials for patients did not increase the outcome of the three Choosing Wisely recommendations for internal medicine in the Netherlands. This could be due to a low adherence of the implementation, since only 19% of the subset of patients in the EDs received the patient summary leaflet. Nearly half of the small sample of patients who read the information about treatment limitations stated that the information helped them in the discussion with the physician. Therefore, we think that the patient information leaflets could still have important value in the conversations between physicians and patients.

Early results of seven Choosing Wisely recommendations showed also only minimal improvement in the USA, and structural outcome evaluations were missing.^3^ Four years later, reminders and patient education handouts for three Choosing Wisely recommendations was only associated with a small and unsustained increase in performance.^13^ Evidence about reducing low-value care is increasing, and recently, a framework was developed to reduce low-value care, which includes rigorous evaluation of Choosing Wisely implementation programmes.^14^ We found that 4 years after the start of the campaign, the implementation of the Choosing Wisely recommendation was already quite good in the EDs. The implementation was outstanding for the second recommendation, where only one patient received an abdominal X-rays for acute abdominal pain. Likewise, recommendation 3 was followed in 92% of all hospitalised patients. So, in these two recommendations, the low-value care was lower than our aim of 15%. However, results of an implementation study in Canada showed an increase in documented orders for treatment limitations, namely, 33% before implementation, 75% during implementation and 100% after 8 months of implementation.^15^ The implementation of the first recommendation was the lowest, with 20% of the patients who received a urinary catheter had no appropriate indication. Although this seems too much, earlier studies show that inappropriate use of urinary catheters is very common. For example, 28% of 649 catheters were placed without appropriate indication in the baseline period of a multifaceted intervention in 34 EDs in the USA.^16^ In the Netherlands, 32% of 324 catheters were inappropriate in non-surgical wards.^8^ So, 20% inappropriate indications for inserting a urinary catheter is not so high, which could be due to the awareness through the campaign and quality improvement projects to lower inappropriate catheter use.

We found no benefit of the patient information leaflets. The mean reason is probably due to the low adherence to the distribution of the leaflets. Further, this could be due to the already high implementation of the recommendations, since it has been shown before that improvement is larger when the baseline performance is poor,^17^ or due to shift changes with many different physicians in the ED. Earlier studies demonstrated that patient information leaflets can be very useful, especially for acute conditions where leaflets also improve adherence to treatment.^18^ A very recent controlled before–after study in two French EDs also showed that patients information leaflets improved communication between physicians and patients, and changed physicians behaviour to better care, since the number of re-consultations reduced from 32% to 18% (OR 0.46; 95% CI 0.27 to 0.77).^19^ In that study, 95% of the patients received an information leaflet and 86% read it. Of course, patient information leaflets will only be useful if patients receive, read and understand information. In our case, our ED seems not to be a workable setting for the implementation of patient materials by nurses or physicians due to the current work overload in EDs in the Netherlands. We do not know whether this intervention would be useful when a special quality healthcare worker would be handing out the leaflets.

### Limitations

There are some limitations to be mentioned. First, although patient information leaflets were electronically distributed and paper forms were available in the EDs, most patients did not receive and read the leaflets. To implement the leaflets as part of regular care, in collaboration with the coordinating research nurse of the ED, we planned to disseminate the leaflets through the nurses in the triage room. Most patients have to wait quite some time to see a physician and for the laboratory results, and we thought that patients could use this time to read the leaflet. However, at the moment, this improvement project started the workload for nurses in the ED was a real problem, since a shortage of qualified nursing staff exists in the Netherlands.^20^ Therefore, the head of the ED of the first hospital stated that the workload of nurses could not be increased by handing over leaflets to patients or by being part of our quality improvement project. In addition, it was not possible to handover the leaflets to the registration/check-in secretary. Therefore, we had to disseminate the leaflets through the residents of the internal medicine instead. In the second hospital, we faced similar problems and also decided to disseminate the leaflets through residents. Residents were reminded to the leaflets through weekly small talks by the study coordinator in the first hospital; in the other hospital, the resident of the quality improvement team was daily present in the ED. For clarity, we asked some internal medicine residents about this process. Thereafter, we speculate that there were three main reasons why residents did not handover the leaflets. First, because they forgot the leaflet during the rushing moments in the ED. Second, because they thought the recommendations were not suitable for their patients. Third, since they already discussed symptoms and treatment options with their patients, they rather discussed the recommendations themselves without using the leaflet. Afterwards, we have to conclude that the project was probably not a priority for the management of the ED. The head of the ED agreed to start the project, but eventually we were unable to collaborate with the ED staff. With hindsight, we should have used different improvement cycles to increase the dissemination of the summary leaflets, with the help of some tools as, for example, a cause-and-effect diagram, a Plan-Do-Study-Act (PDSA) form, and/or run chart to let the quality improvement team reflect on the process.

The second limitation was the academic setting, with two EDs of university medical centres. Although the three recommendations are applicable in all hospitals in the Netherlands, we cannot extrapolate the results to non-university hospitals.

Next, if treatment limitations were discussed but not reported, we scored this as not reported. Although this could result in an underestimation of reported treatment limitations, this is in accordance with clinical practice. If physicians do not report treatment limitations, in practice this means that healthcare workers see this as ‘No treatment limitations’.

Further, we only focused on quantitative data since we aimed to implement the Choosing Wisely recommendations for internal medicine in two EDs. We could not include a detailed process evaluation of this quality improvement project. Further, we have no qualitative data of patients and physician experiences.

Finally, the power dynamic between patients and their physician, which is usually higher in a stressful setting as the ED, could potentially impact the results of our patient leaflets.

### Strengths

This improvement project has some important strengths and implications. We did this improvement project in real practice and learnt that our ED is not the best setting to disseminate patient information leaflets, mainly due to a lack of management engagement and a shortage staff. Although the implementation of the three recommendations for internal medicine did not increase, the patients who received the information about treatment limitations reported that this helped them in the conversation with their physician. Most discussions about treatment limitations are in fact about cardiopulmonary resuscitation. However, the discussion around ‘Do not attempt cardiopulmonary resuscitation’ is difficult and often delayed. A review from 2016 found that physicians often hesitate to start this conversation due to concerns about possible distress for patients and fears of complaints.^21^ Furthermore, patients will not initiate the conversations themselves, even though they are willing to discuss treatment limitations,^22^ but our patient information leaflet could help to start this conversation. Next, this intervention is simple, low-cost and part of regular care, therefore well suited for further dissemination. To promote conversation between physicians and patients, future quality improvement projects could just use the patient information leaflet about treatment limitations instead of the summary leaflet of the three recommendations, since nearly half of the patients were helped by this leaflet. Furthermore, since the dissemination of leaflets was difficult, other non-printed formats to deliver patient information could be through multimedia, such as videos, audio records, patient-focused podcasts or web-based tools. However, no clear effect difference between print and multimedia has been demonstrated.^23^

## Conclusion

We provided simple, low-cost educational materials for patients as part of regular care. However, probably due to a lack of dissemination of the summary leaflets and the good baseline scores, this did not result in better implementation of the three recommendations for internal medicine in the ED. A workplace with a lack of management engagement due to shortage of staff is no useful setting for improvement projects. The patient information leaflet in itself could be a useful tool to start the difficult conversation about treatment limitations.
