# Supplementary material for: Patient education materials to implement choosing wisely recommendations for internal medicine at the emergency department
Source: BMJ Open Qual. 2021 Feb 4;10(1):e000971. doi: 10.1136/bmjoq-2020-000971 (PMC7871247; doi:10.1136/bmjoq-2020-000971)
Supplement: Supplementary data [file bmjoq-2020-000971supp001.pdf]

Verstandige Keuzes van de Interne Geneeskunde op de Spoedeisende Hulp

U heeft zich gemeld bij de spoedeisende hulp van ons ziekenhuis. In het kader van de landelijke campagne Verstandig Kiezen informeren wij u in deze folder over drie verstandige keuzes. Als u nog vragen heeft, stel deze dan gerust aan uw arts of verpleegkundige.

1. Een blaaskatheter is niet nodig wanneer u niet ernstig ziek bent, en zelf nog kunt plassen

Een blaaskatheter is een slangetje in de blaas om de urine weg te laten lopen. Het plaatsen van een blaaskatheter brengt risico's met zich mee, bijvoorbeeld een verhoogde kans op een urineweginfectie. Dit is een vaak voorkomend probleem in Nederlandse ziekenhuizen. Kunt u zelf nog goed plassen? Vraag uw arts dan of het voor uw behandeling echt noodzakelijk is dat u een blaaskatheter krijgt.
Voor meer informatie kunt u naar de AMC- website voor de folder 'Blaaskatheter (via de plasbuis) tijdens een opname'.

2. Een röntgenfoto van de buik is niet nodig voor acute buikpijn

Een buikoverzichtsfoto is een röntgenfoto van de buik. Er worden foto's gemaakt door middel van röntgenstraling om een algemene indruk van de buikorganen te krijgen. Het geeft bij acute buikpijn niet voldoende informatie. Een goede anamnese (de ziektegeschiedenis die u aan de dokter vertelt), lichamelijk onderzoek en laboratoriumonderzoek geven meer informatie. Daarom is het bij buikpijn onnodig om een buikoverzichtsfoto van de buik te maken.

3. Bespreek eventuele behandelbeperkingen met uw dokter

Als patiënt komt u op de spoedeisende hulp met een bepaalde behandelwens. Meestal is dit het genezen van ziekte of het verlichten van uw klachten. Wij geven u de best beschikbare behandeling om dit te bereiken.
Er zijn misschien ook grenzen die u stelt aan uw behandeling. Het afzien van een bepaalde behandeling, bijvoorbeeld niet gereanimeerd worden, noemen wij een behandelbeperking. In het ziekenhuis is het de regel dat wij alles doen om u te behandelen, behalve als er een behandelbeperking is geregistreerd. Uw arts kan ook initiatief nemen tot een behandelbeperking. Dit gebeurt als een behandeling niet (meer) medisch zinvol is.
Meer informatie kunt u lezen in de folder 'Praten over behandelwensen en -grenzen, informatie voor patiënten en familie'.

Heeft u nog vragen?

Stel deze dan gerust aan uw arts of verpleegkundige.

Afdeling Interne Geneeskunde / Patiëntenvoorlichting

IDnummer: 000059325, Versie: 1, Publicatiedatum: 5-7-2018
