# Supplementary material for: Patient education materials to implement choosing wisely recommendations for internal medicine at the emergency department
Source: BMJ Open Qual. 2021 Feb 4;10(1):e000971. doi: 10.1136/bmjoq-2020-000971 (PMC7871247; doi:10.1136/bmjoq-2020-000971)
Supplement: Supplementary data [file bmjoq-2020-000971supp002.pdf]

Blaaskatheter (via de plasbuis) tijdens een opname

U bent opgenomen in het ziekenhuis, en u heeft of krijgt een blaaskatheter. In deze folder leest u informatie over de blaaskatheter. Als u nog vragen heeft, stel deze dan gerust aan uw arts of verpleegkundige.

Wat is een blaaskatheter?

Een katheter is een soepele, holle slang. Bij een blaaskatheter brengt een verpleegkundige of een arts dit slangetje via de plasbuis (urethra) in de blaas . De urine uit de blaas wordt opgevangen in een urinezak. De blaaskatheter blijft zitten door een ballonnetje dat wordt gevuld met water, zie onderstaande figuur.

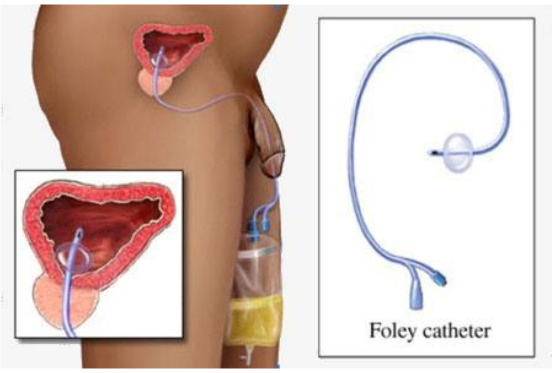

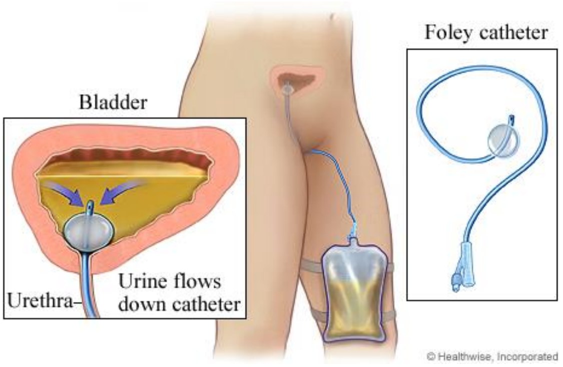

Hoe gaat het inbrengen van een blaaskatheter?

Een arts of een verpleegkundige brengt de katheter in. U ligt tijdens het inbrengen van de katheter op uw rug. De arts of verpleegkundige spuit een glijmiddel met verdovende werking in de plasbuis (urethra). Hierna kan hij of zij de katheter inbrengen. De katheter wordt met behulp van een ballonnetje 'gefixeerd' en aangesloten op een katheterzak.
Na het inbrengen van de katheter kunt u soms het gevoel hebben alsof u moet plassen. Dit is een reactie van de blaas op de katheter. Dit noemen wij ook wel 'loze aandrang'.

Hoe houdt u de katheter schoon?

Met een katheter kunnen bacteriën gemakkelijker in uw blaas en urinewegen komen. Om zo goed mogelijk te voorkomen dat er een urineweginfectie ontstaat, is het nodig om dagelijks de plaats waar de katheter naar binnen gaat, schoon te maken. U kunt dit doen met een washandje met gewoon leidingwater. U hoeft niet bang te zijn dat de katheter er uit zal vallen als u er aan komt, deze zit immers met het gevulde ballonnetje in de blaas. Voor u gaat schoon maken moet u goed uw handen wassen. Gebruik per wasbeurt een schoon washandje.

Bij mannen: Schuif de voorhuid van de penis naar achteren, reinig de eikel en de directe omgeving met een natte washand. Spoel het washandje telkens uit met leidingwater. Maak ook de ingangsplaats van de katheter schoon met de washand. Schuif na reiniging de voorhuid weer terug. Uiteraard kunt u ook tijdens het douchen de eikel goed schoonspoelen.

Bij vrouwen: Spreid met een hand de schaamlippen en maak met een nat washandje de uitgang van de plasbuis en de vagina goed schoon. Spoel het washandje telkens uit met leidingwater. Reinig u van voren naar achter. Maak ook de ingangsplaats van de katheter schoon met de washand. Als u onder de douche gaat kunt u ook met de douchekop de vagina goed schoon spoelen.

Gebruik geen crème of talkpoeder in de buurt van de plasbuis.

Hoe lang blijft de urinekatheter zitten?

De tijd dat de blaaskatheter blijft zitten is afhankelijk van de reden voor het inbrengen van de blaaskatheter. De meest voorkomende reden om een katheter in te brengen is **urineretentie**. Bij urineretentie kunt u niet meer goed uitplassen, er blijft te veel urine achter. De blaaskatheter helpt dan de urine af te voeren uit de blaas. De katheter kan eruit wanneer u weer normaal kunt plassen.

Een andere vaak voorkomende reden voor het inbrengen van een blaaskatheter op de Spoedeisende Hulp (SEH) of de afdeling is het **monitoren van de urineproductie bij een ernstig zieke patiënt**. Als u ernstig ziek bent, is het voor de arts en verpleegkundige belangrijk om de hoeveelheid urine nauwkeurig (ieder uur) in de gaten te houden. De urineproductie zegt iets over de doorbloeding van de organen (in dit geval de nieren). Meestal bent u na 2 tot 3 dagen weer voldoende opgeknapt, en kan de urinekatheter weer worden verwijderd. Dit kunt u ook met uw arts of verpleegkundige bespreken.

Heeft u een andere reden voor het inbrengen van de blaaskatheter, of weet u niet waarom u nog een blaaskatheter heeft. Vraag dan uw arts of verpleegkundige naar de reden, en hoe lang u deze nog nodig heeft.

Leefregels en adviezen

- Leeg de opvangzak regelmatig in het toilet, wacht niet tot de zak (over)vol zit.
- Hang de katheterzak lager dan de blaas, anders kan de urine niet aflopen, en zorg dat de katheterzak geen contact maakt met de vloer.
- Probeer elke dag een douche te nemen, hierbij kunt u uw beenzak aanhouden.
- Vermijd het dragen van strakke kleding. De afvoerslang mag niet geknikt of afgekneld zijn.
- Drink goed! Van alles wat u drinkt, komt ongeveer tweederde in de blaas terecht. Dit is afhankelijk van het seizoen en uw activiteiten. Misschien merkt u dat u de neiging heeft om minder te gaan drinken, omdat u een katheter heeft. Doet u dit niet. Het is verstandig om per 24 uur ongeveer 1,5 liter urine te produceren, behalve als uw arts anders adviseert.

Laan BJ, *et al. BMJ Open Qual* 2021; 10:e000971. doi: 10.1136/bmjoq-2020-000971

Klachten door de blaaskatheter

Het kan voorkomen dat er urine lekt via de plasbuis langs de katheter. Dit komt meestal door **blaaskrampen**. Een katheter is een lichaamsvreemd voorwerp en de blaas reageert hierop met samentrekken. Vertel het aan uw arts als u veel last heeft van de blaaskrampen. De arts kan eventueel medicijnen voorschrijven om urineverlies door de blaaskrampen tegen te gaan.

Verder heeft u met een blaaskatheter een verhoogde kans op een **urineweginfectie**. Het standaard controleren van de urine heeft geen zin, aangezien er bijna altijd bacteriën op een blaaskatheter aanwezig zijn. Klachten bij een urineweginfectie / blaasontsteking zijn onder andere koorts, stinkende urine of een zwelling van de bijbal. Heeft u klachten die passen bij een urineweginfectie, overleg dit dan met uw arts.

Wanneer belt u de verpleegkundige?

- Als u aanhoudende pijn heeft.
- Als de kleur van de urine plotseling (donker)rood wordt door bloed bij de urine.
- Als er 2 tot 3 uur geen urine in de zak komt. Ga eerst na of er een knik in de slang zit, of u genoeg gedronken heeft en of de katheterzak niet te hoog hangt.
- Als u blijvende lekkage langs de katheter heeft.

Blaaskatheter mee naar huis

Vraag uw verpleegkundige naar de folder ‘Blaaskatheter (via de plasbuis) en de verzorging van de blaaskatheter thuis’.

Heeft u nog vragen?

Stel deze dan gerust aan uw arts of verpleegkundige.

Interne Geneeskunde / Patiëntenvoorlichting

Laan BJ, *et al. BMJ Open Qual* 2021; 10:e000971. doi: 10.1136/bmjoq-2020-000971
