# Supplementary material for: Patient education materials to implement choosing wisely recommendations for internal medicine at the emergency department
Source: BMJ Open Qual. 2021 Feb 4;10(1):e000971. doi: 10.1136/bmjoq-2020-000971 (PMC7871247; doi:10.1136/bmjoq-2020-000971)
Supplement: Supplementary data [file bmjoq-2020-000971supp003.pdf]

Praten over behandelwensen en –grenzen, informatie voor patiënten en familie

Inleiding

Als patiënt komt u in het AMC met een bepaalde behandelwens.
Meestal is dit het voorkomen of genezen van ziekte of het verlichten van uw klachten. Wij geven u de best beschikbare behandeling om dit te bereiken. Misschien zijn er ook grenzen die u stelt aan uw behandeling in het ziekenhuis. Sommige patiënten willen bijvoorbeeld niet gereanimeerd worden.
Het afzien van een bepaalde behandeling noemen we een 'behandelbeperking'. Uw arts kan ook initiatief nemen tot een behandelbeperking. Dit gebeurt als een behandeling niet (meer) medisch zinvol is. Met deze folder willen wij u voorbereiden op een gesprek over behandelbeperkingen. Deze folder is bedoeld voor wilsbekwame volwassenen. Hebt u na het lezen van de folder nog vragen over dit onderwerp? Dan kunt u uiteraard terecht bij uw behandelend arts.

Waarom praten over behandelbeperking?

Het is belangrijk om uw wensen en verwachtingen over behandelbeperkingen kenbaar te maken bij uw behandelend arts. Uw arts bespreekt dan met u wat de mogelijkheden en onmogelijkheden van een behandeling zijn. Dit om te voorkomen dat u in een acute situatie een emotioneel gesprek met een andere, voor u onbekende arts moet voeren. Een behandelbeperking wordt altijd met u afgestemd.

Welke behandelbeperkingen zijn er?

- Niet reanimeren: dit is een belangrijke en meest voorkomende behandelbeperking. Reanimeren is een poging om de bloedsomloop en de ademhaling weer te herstellen. Voor meer uitleg over reanimeren, zie het kader verderop in de folder.
- Niet beademen: de ademhaling niet kunstmatig overnemen met een beademingsmachine. Beademen gebeurt op een intensive care.
- Niet naar IC (intensive care): geen opname op de intensive care voor intensieve zorg, bijvoorbeeld voor ondersteuning van de bloeddruk of voor kunstmatige beademing.
- Niet naar CCU (hartbewaking): geen opname op de afdeling hartbewaking voor bewaking van het hartritme.
- Geen dialyse: geen vervanging van de nierfunctie.
- Geen operaties.
- Geen bloedproducten: geen toediening van rode bloedcellen of bloedplaatjes.
- Geen antibiotica: geen behandeling van bacteriële infecties.
- Geen sondevoeding: geen toediening van kunstmatige voeding via (maag)sonde.
- Geen invasieve diagnostiek: geen nieuwe onderzoeken die belastend zijn voor de patiënt.

Laan BJ, *et al. BMJ Open Qual* 2021; 10:e000971. doi: 10.1136/bmjoq-2020-000971

- Geen verdere behandeling: het stoppen van alle behandelingen, behalve behandelingen die gericht zijn op comfort, zoals bestrijding van pijn en benauwdheid. Het gaat hier niet om actieve levensbeëindiging (euthanasie).

Dit zijn de belangrijkste behandelbeperkingen. De eerste drie (niet reanimeren, niet beademen, niet naar IC) bespreekt en registreert de behandelend arts bij iedere patiënt die wordt opgenomen in het ziekenhuis.

Wanneer praten over behandelbeperking?

Tijdens een opname in het ziekenhuis kan het gebeuren dat u plotseling in een medische noodsituatie komt, waarbij een ingrijpende behandeling of zelfs reanimatie nodig is. Omdat in een acute situatie meestal geen tijd is om hierover te praten, is het verstandig om al eerder na te denken en te praten over behandelbeperkingen. De precieze timing hiervan bepaalt u of uw arts. U kunt altijd zelf aangeven dat u een bepaalde behandelbeperking wilt laten registreren in uw elektronisch patiënten dossier (EPD). Uw arts kan ook het initiatief nemen om met u over behandelbeperkingen te praten. Dit gebeurt in ieder geval bij een opname in het ziekenhuis, maar dit kan ook tijdens een poliklinische afspraak. Afspraken over de behandelbeperking kunt u herzien en opnieuw bespreken met uw arts, bijvoorbeeld als uw gezondheidstoestand of uw wensen zijn veranderd.

Wie besluit tot een behandelbeperking?

- Uzelf: als u zelf niet wilt dat bepaalde handelingen worden toegepast. Bijvoorbeeld door een bepaalde religie/levensbeschouwing of omdat u geen belastende behandelingen (meer) wilt ondergaan. Ook kan de best haalbare uitkomst van een behandeling voor u niet aanvaardbaar zijn, bijvoorbeeld vanwege een grote kans op blijvende schade of omdat er geen uitzicht is op volledig herstel of herstel tot een acceptabel niveau. Het is belangrijk om dit met uw behandelend arts te bespreken.
- Uw arts: sommige patiënten zijn zo ziek of verzwakt, dat bepaalde behandelingen zoals reanimatie of behandeling op de intensive care geen kans van slagen hebben en daarom medisch niet zinvol zijn. Deze behandelingen worden dan niet toegepast, ook niet als de patiënt dit wel wenst. Indien mogelijk bespreekt uw arts met u een dergelijke situatie tijdig en registreert dit nauwkeurig.

Wat doet het AMC met uw besluit?

De arts legt de afgesproken behandelbeperking(en) en eventuele wijzigingen vast in het elektronisch patiënten dossier (EPD). Alle zorgverleners binnen het AMC kunnen dit dossier raadplegen. Wat u op de polikliniek hebt afgesproken, is op deze manier ook zichtbaar als u wordt opgenomen. De afspraken gelden alleen binnen het AMC, maar worden vaak ook vermeld aan de huisarts.

Hebt u vragen?

We hopen dat de informatie in deze folder u meer duidelijkheid heeft gegeven over het nut van praten over behandelbeperkingen. Het onderwerp kan moeilijk zijn voor u en uw familie, maar ook voor de betrokken zorgverleners. Het is belangrijk om goed over het onderwerp en uw beslissing(en) na te denken en te bespreken met uw behandelend arts. Natuurlijk kunt u met uw vragen altijd terecht bij uw behandelend arts.

Meer informatie over reanimatie

Tijdens een opname is er altijd een zeer kleine kans dat bij een patiënt de bloedsomloop of ademhaling stopt. In het AMC is het de regel dat een patiënt in dat geval altijd gereanimeerd wordt, behalve als er een niet reanimeren beleid is geregistreerd.

Wat is reanimeren?

Reanimeren is een poging tot herstel van de bloedsomloop en ademhaling als deze levensbedreigend verstoord zijn. Reanimatie wordt toegepast als de ademhaling of hartslag plotseling tot stilstand komt. Bij zo'n acute situatie is snel handelen nodig, omdat er anders, door zuurstofgebrek, beschadiging van de hersenen optreedt. Bij een reanimatie proberen de artsen en verpleegkundigen met hartmassage, beademing, elektrische schokken en medicijnen de bloedsomloop en de ademhaling kunstmatig op gang te krijgen.
Binnen het ziekenhuis is een speciaal reanimatieteam op afroep beschikbaar. Als een patiënt een adem- of hartstilstand krijgt, is dit team snel ter plaatse.

Wat is het resultaat van reanimatie?

Een reanimatie kan levensreddend zijn, maar helaas is niet vooraf te voorspellen wie na reanimatie zal overleven. Ook als een reanimatie succesvol is, is er een reële kans op het ontstaan van schade aan de hersenen door zuurstofgebrek. De kwaliteit van leven is dan niet vergelijkbaar met de situatie voorafgaand aan de reanimatie. De kans van slagen hangt sterk af van de oorzaak van de adem- of hartstilstand, de leeftijd en de conditie. Uw arts kan u hierover verder informeren. Echter, niemand kan met zekerheid de uitkomst van een reanimatie voorspellen.

Wel of niet reanimeren?

De kans dat een plotselinge adem- of hartstilstand optreedt, is over het algemeen zeer klein. Toch kan het voorkomen. Als een patiënt niet wordt gereanimeerd, komt hij of zij bij een hart- of ademstilstand te overlijden. Binnen het AMC is de afspraak dat iedere patiënt die een hart- of ademstilstand krijgt gereanimeerd wordt, tenzij:

1. *De patiënt heeft besloten en laten vastleggen geen reanimatie te willen. Dit besluit is dan van tevoren besproken met de behandelend arts en genoteerd als behandelbeperking 'niet reanimeren' in het elektronisch patiënten dossier (EPD).* ***Als u beschikt over een wilsverklaring of niet-reanimeren penning, geef dit dan aan bij uw behandelend arts, zodat deze informatie in het elektronisch patiënten dossier kan worden vastgelegd.***
2. *Er medische redenen zijn om af te zien van een reanimatie. Bij sommige patiënten besluit de behandelend arts dat een reanimatie, beademing of behandeling op de intensive care niet medisch zinvol is, bijvoorbeeld bij patiënten op zeer hoge leeftijd en/of met ernstige aandoeningen. Dit wordt met de patiënt en zijn of haar familie bij opname in het ziekenhuis (of eerder) besproken en vastgelegd in het elektronisch patiënten dossier (EPD).*

Afdeling Interne Geneeskunde / Patiëntenvoorlichting
