# Supplementary material for: Patient education materials to implement choosing wisely recommendations for internal medicine at the emergency department
Source: BMJ Open Qual. 2021 Feb 4;10(1):e000971. doi: 10.1136/bmjoq-2020-000971 (PMC7871247; doi:10.1136/bmjoq-2020-000971)
Supplement: Supplementary data [file bmjoq-2020-000971supp004.pdf]

S4 Figure. Result Choosing Wisely recommendation 1 per month.

(1) Do not place an indwelling urinary catheter in non-critically ill patients who can void

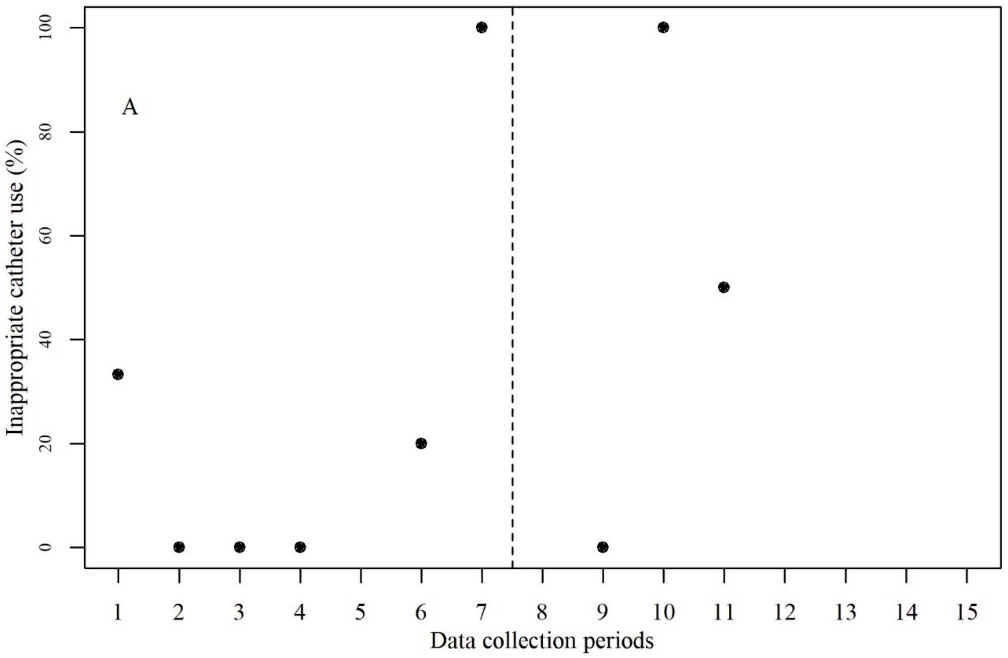

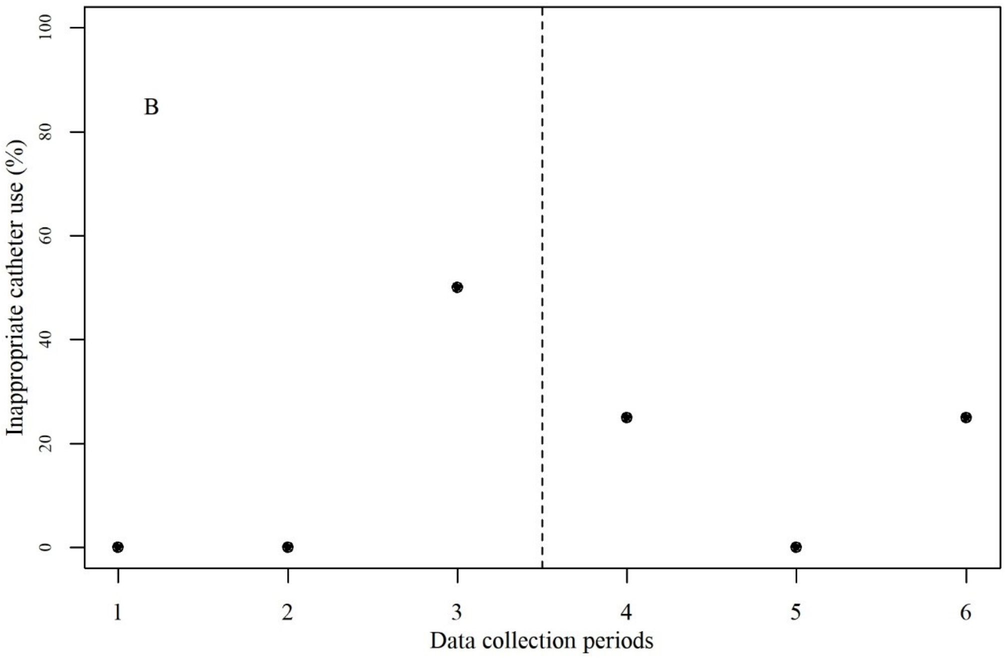

A=University hospital 1. B=University hospital 2.
