# Supplementary material for: Patient education materials to implement choosing wisely recommendations for internal medicine at the emergency department
Source: BMJ Open Qual. 2021 Feb 4;10(1):e000971. doi: 10.1136/bmjoq-2020-000971 (PMC7871247; doi:10.1136/bmjoq-2020-000971)
Supplement: Supplementary data [file bmjoq-2020-000971supp005.pdf]

S5 Figure. Result Choosing Wisely recommendation 3 per month.

(3) Discuss whether treatment limitations are needed when talking to patients about treatment options

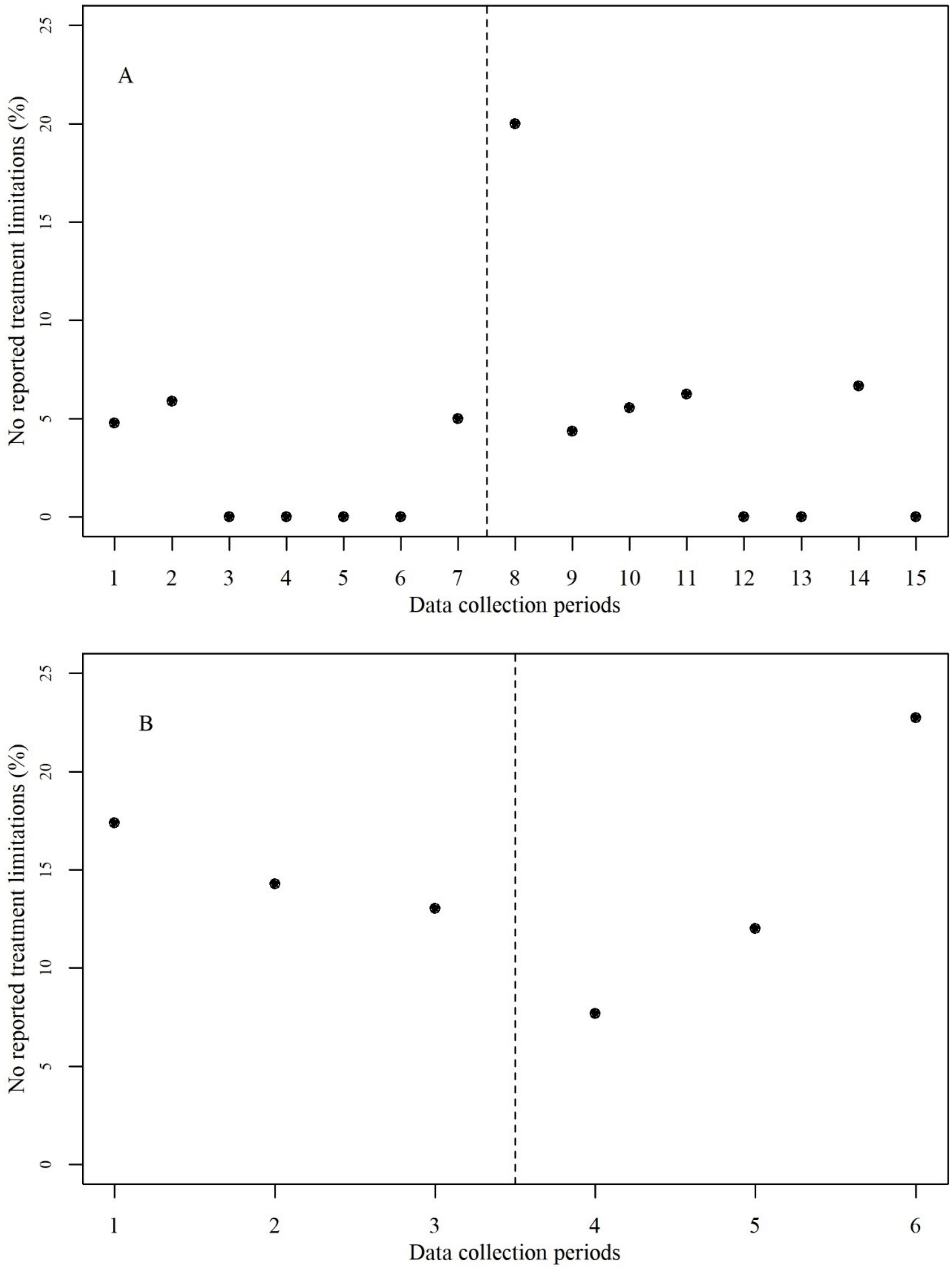

A=University hospital 1. B=University hospital 2.
