# Supplementary material for: Patient education materials to implement choosing wisely recommendations for internal medicine at the emergency department
Source: BMJ Open Qual. 2021 Feb 4;10(1):e000971. doi: 10.1136/bmjoq-2020-000971 (PMC7871247; doi:10.1136/bmjoq-2020-000971)
Supplement: Supplementary data [file bmjoq-2020-000971supp006.pdf]

S1 Table. Adjusted analysis of Choosing Wisely recommendation 1.

(1) Do not place an indwelling urinary catheter in non-critically ill patients who can void

Variable	Beta intervention*	Bivariable OR (95% CI)	*P* value	Multivariable OR (95% CI)	*P* value
Intervention*	0.427	1.533 (0.346−6.794)	0.574	3.197 (0.322−31.794)	0.321
Age	0.383	1.062 (0.988−1.140)	0.102	1.075 (0.987−1.170)	0.098
Male Sex	0.561	1.724 (0.347−8.554)	0.505	2.478 (0.345−17.812)	0.367
Hospital	0.902	0.223 (0.035−1.407)	0.110	0.101 (0.009−1.092)	0.059
Specialism	0.286	1.192 (0.759−1.871)	0.445	1.254 (0.629−2.499)	0.520
Charlson comorbidity index	0.376	1.192 (0.256−5.560)	0.823	0.456 (0.052−3.962)	0.477
High MEWS score	0.451	0.689 (0.120−3.946)	0.675		
Hospitalized 3 mo before ED visit	0.386	1.266 (0.280−5.733)	0.760		
Outpatient care for internal medicine 1 year before presentation at the ED	0.273	0.617 (0.293−7.934)	0.617	3.466 (0.325−37.011)	0.999
Nursing home resident	0.375	NA	0.999		0.999
Hospitalization	0.470	NA	0.999		0.999

Variables showing a difference of more than 10% in beta for baseline and intervention period were included in the multivariate logistic models to adjust for confounding.
*Intervention is the difference between the baseline and intervention period.
