# Supplementary material for: Patient education materials to implement choosing wisely recommendations for internal medicine at the emergency department
Source: BMJ Open Qual. 2021 Feb 4;10(1):e000971. doi: 10.1136/bmjoq-2020-000971 (PMC7871247; doi:10.1136/bmjoq-2020-000971)
Supplement: Supplementary data [file bmjoq-2020-000971supp007.pdf]

S2 Table. Adjusted analysis of Choosing Wisely recommendation 3.

(3) Discuss whether treatment limitations are needed when talking to patients about treatment options

**Variable**	**Beta intervention***	**Bivariable OR (95% CI)**	***P* value**	**Multivariable OR (95% CI)**	***P* value**
Intervention*	0.340	1.405 (0.663−2.978)	0.374	1.431 (0.659−3.107)	0.365
Age	0.354	0.995 (0.975−1.015)	0.602		
Male Sex	0.333	0.390 (0.182−0.835)	0.015		
Hospital	0.282	3.967 (1.799−8.751)	0.001	4.610 (2.060−10.316)	<0.001
Specialism	0.336	1.015 (0.789−1.306)	0.906		
Charlson comorbidity index	0.347	1.092 (0.509−2.343)	0.821		
High MEWS score	0.326	0.834 (0.240−2.891)	0.774		
Hospitalized 3 mo before ED visit	0.387	0.390 (0.155−0.980)	0.045	0.308 (0.120−0.793)	0.015
Outpatient care for internal medicine 1 year before presentation at the ED	0.354	0.424 (0.196−0.919)	0.030		
Nursing home resident	0.341	0.841 (0.107−6.638)	0.870		
